# Preferences for pre-exposure prophylaxis among U.S. military men who have sex with men: results of an adaptive choice based conjoint analysis study

**DOI:** 10.1186/s40779-021-00323-6

**Published:** 2021-05-19

**Authors:** José I. Gutierrez, Alex Dubov, Frederick L. Altice, David Vlahov

**Affiliations:** 1 Yale School of Nursing, 400 West Campus Drive, Orange, CT 06477 USA; 2 Philip R. Lee Institute for Health Policy Studies, University of California, San Francisco, 3333 California St, San Francisco, CA 94118 USA; 3 Loma Linda University, Griggs Hall 11065, Loma Linda, CA 92350 USA; 4 Yale School of Medicine, Section of Infectious Diseases, 135 College Street, Suite 323, New Haven, CT 06510 USA; 5 Department of Epidemiology-Microbial Diseases, Yale School of Public Health, 60 College St, New Haven, CT 06510 USA

**Keywords:** Conjoint analysis, Pre-exposure prophylaxis, Preference, Decision science, HIV, Military health, Infectious disease

## Abstract

**Background:**

Pre-exposure prophylaxis (PrEP) prevents human immunodeficiency virus (HIV) infection, but its use remains low among U.S. military men who have sex with men (MSM), likely due to mis-matching with personal preferences. We conducted a study to characterize preferences to PrEP measures within this population.

**Methods:**

HIV-negative military MSM were recruited through a closed, Lesbian, Gay, Bisexual, and Transgendered (LGBT) military social media group. The survey was anonymous, and consisted of five experimentally varied attributes in service delivery: dosing method, provider type, visit location, lab work evaluation location, and dispensing venue. Relative importance and part-worth utility scores were generated using hierarchical bayes (HB) estimation, and the randomized first choice model was used to examine participation interest across eight possible PrEP program scenarios.

**Results:**

A total of 429 participants completed the survey. Among the eight scenarios with varying attributes, the most preferred scenario featured a daily tablet, PrEP injection or implant, along with a military provider, smartphone/telehealth visit, and on-base locations for lab evaluation and medication pick-up. The results also emphasized the importance for providers to be familiar with PrEP prescription knowledge, and to provide interactions sensitive to sexual identity and mental health.

**Conclusion:**

A PrEP program consisting of daily tablet is preferred in military healthcare settings is preferred. Long-acting implants and injections are also desired.

**Supplementary Information:**

The online version contains supplementary material available at 10.1186/s40779-021-00323-6.

## Background

Each year, there are approximately 350 new cases of human immunodeficiency virus (HIV) infections within members of the U.S. military; with those most affected being younger, Black, and men who have sex with men [1–6]. When taken as prescribed, pre-exposure prophylaxis (PrEP) effectively prevents HIV infection [7–10]. However, the percentage of at-risk military members taking PrEP measures is only 16%, and even lower in members of color [2].

Factors that contribute to the low PrEP engagement rate include access pathways, delivery mechanisms, and dosing methods impacted by geographic, psychological, and adherence barriers [11–18]. For military members, PrEP availability largely depends on geographic proximity to large medical facilities with specialty services, as evidenced by 41% of all military PrEP prescriptions originating from military medical centers in three locations in the U.S. Additionally, 60% of all military PrEP prescriptions also occur only after consultation with an infectious disease specialist [2]. Features of PrEP delivery programs suitable for military MSM remain unknown.

Health services designed around preferences in terms of product type, delivery method, and location settings have been shown to produce improved outcomes and retention in care [19–21]. Stated preference methods, such as conjoint analysis, quantify preference data of new market entrants and product attributes [16, 17, 22–26]. In conjoint analysis, products/programs are viewed as a composition of various attributes that possess a certain amount of value (part-worth utility score) determined by preference. By quantifying these part-worth utility scores for preferred attributes, these scores can then be entered into market simulation models to predict how respondents might respond to *any* potential combination of attribute levels [16, 17, 22–26]. We used conjoint analysis to identify preferred attributes that are most influential to at-risk U.S. military MSM’s decision to take PrEP within the military healthcare system.

## Methods

### Data collection

A convenience sample of self-reported HIV-negative, U.S. military MSM and trans-individuals were recruited between March and April 2020 through a closed Facebook group with over 7000 LGBT U.S. military members. The group administrators placed monthly advertisements describing the study on the group’s main forum. Those interested could click on a link to access an online study disclosure form with a ‘click to consent’ procedure. An option to provide an e-mail address that was not linked to survey responses was offered to participants who opted to receive $5 compensation for questionnaire completion. The study was approved by the Yale University Institutional Review Board.

To collect and quantify respondent preference data, an adaptive choice-based conjoint (ACBC) survey instrument was developed based on a starting set of PrEP program attributes resulting from review of the literature of previous PrEP preference conjoint experiments, and then refined through in-depth, qualitative interviews between PrEP experts and U.S. military MSM [2–5, 11, 12, 14, 15, 27–39]. The final survey instrument focuses on modifiable PrEP program characteristics, and consists of the following five attributes: dosing method [daily oral tablet, on-demand tablet regimen (two tablets before sex, one tablet for two days after), rectal douche (before sex), injection (every 2 months), implant (once a year)], provider type (military, civilian), visit location (on-base, off-base, smartphone app), dispensing venue (on-base, off-base, mail delivery), and lab evaluation (on-base, off-base, home-based mail kit). The survey was piloted by the author (JIG) using a convenience sample of eleven military MSM members within the targeted social media group for concept testing, and the descriptions and wording of three attribute categories and two attribute level choices were revised for clarification based on feedback. Additional figure shows a sample item of the conjoint survey, and Table 1 describes the program attributes in the survey. Additional information that we collected include age, race, ethnicity, rank type (officer, enlisted or warrant officer), military branch, geographic region, PrEP experience [“Have you ever used PrEP (Pre-Exposure Prophylaxis)?”], depressive symptoms with the Patient Health Questionnaire-2 (PHQ2) [40, 41], and the HIV Incidence Risk Index for MSM (HIRI-MSM), which identifies MSM at high risk [42]. Measures to explore levels of satisfaction with a current level of HIV protection and disclosure discomfort within interactions with a primary care provider were also collected.

**Table 1:** Description of conjoint survey attributes and associated level options presented to respondents

Attributes and levels	Survey description
Dosing method	
Daily oral tablet	Daily oral tablet means that you would have to take an oral tablet every day (daily) to maintain a protective level of PrEP medication
PrEP injection	PrEP injection means that you get an injection or shot that would provide a protective level of PrEP medication for 2 months
PrEP implant	PrEP implant means that you would get a small implant that would slowly release a protective level of PrEP medication for at least a year
On-demand regimen	On-demand regimen means you take two tablets 24 h before sex and then one tablet daily for the next two days. This dosing method would protect you from HIV for that single sexual encounter only
PrEP rectal douche	Rectal PrEP douche means that you would use a rectal douche or enema prior to having sex that leaves behind protective level of PrEP medication for that sexual encounter
Provider type	
Military	Military provider means that you prefer a medical visit with a healthcare provider that is a member of the military
Civilian	Civilian provider means that you prefer a medical visit with a healthcare provider that is a civilian or not a member of the military
PrEP visit location	
Smartphone	Smartphone/mobile app visit means that you prefer to have a virtual medical visit with a healthcare provider through a smartphone call or mobile app
On-base	On-base medical visit means that you prefer an in-person medical visit with a healthcare provider that is in a clinic on-base
Off-base	Off-base medical visit means that you prefer an in-person medical visit with a healthcare provider that is in a clinic off-base
Lab evaluation location	
Provide labs on-base	Provide lab work on-base means that you prefer to do you lab work at a laboratory or clinic on-base
Provide labs off-base	Provide lab work off-base means that you prefer to do you lab work at a laboratory or clinic off-base
Home-based mail-in kit	Home-based mail-in kit means that you prefer to receive a home-based lab testing kit in the mail. You will provide self-collected, small samples of blood and urine and mail the kit back to the laboratory for evaluation. Your PrEP provider would then see the lab results after processing
PrEP dispensing venue	
Receive PrEP on-base	Receive PrEP on-base means that you prefer to pick up or receive your PrEP medication from a pharmacy/clinic on-base
Receive PrEP off-base	Receive PrEP off-base means that you prefer to pick up or receive your PrEP medication from a pharmacy/clinic off-base
Receive PrEP by mail	Receive PrEP by mail delivery means that you prefer to receive your PrEP medication in the mail at your home or APO

*PrEP* pre-exposure prophylaxis, *APO* Army Post Office

### Data analysis

The final survey was loaded into Lighthouse Studio 9, and an experimental design module was used to pre-test the design with 500 simulated respondents for optimal choice task configuration. The final design produced a survey where each level within an attribute was seen at least three times per respondent; achieving a high degree of precision at the individual level with a standard of error of < 0.03 and all efficiencies reporting at 1.00 [43].

**Fig. 1:**
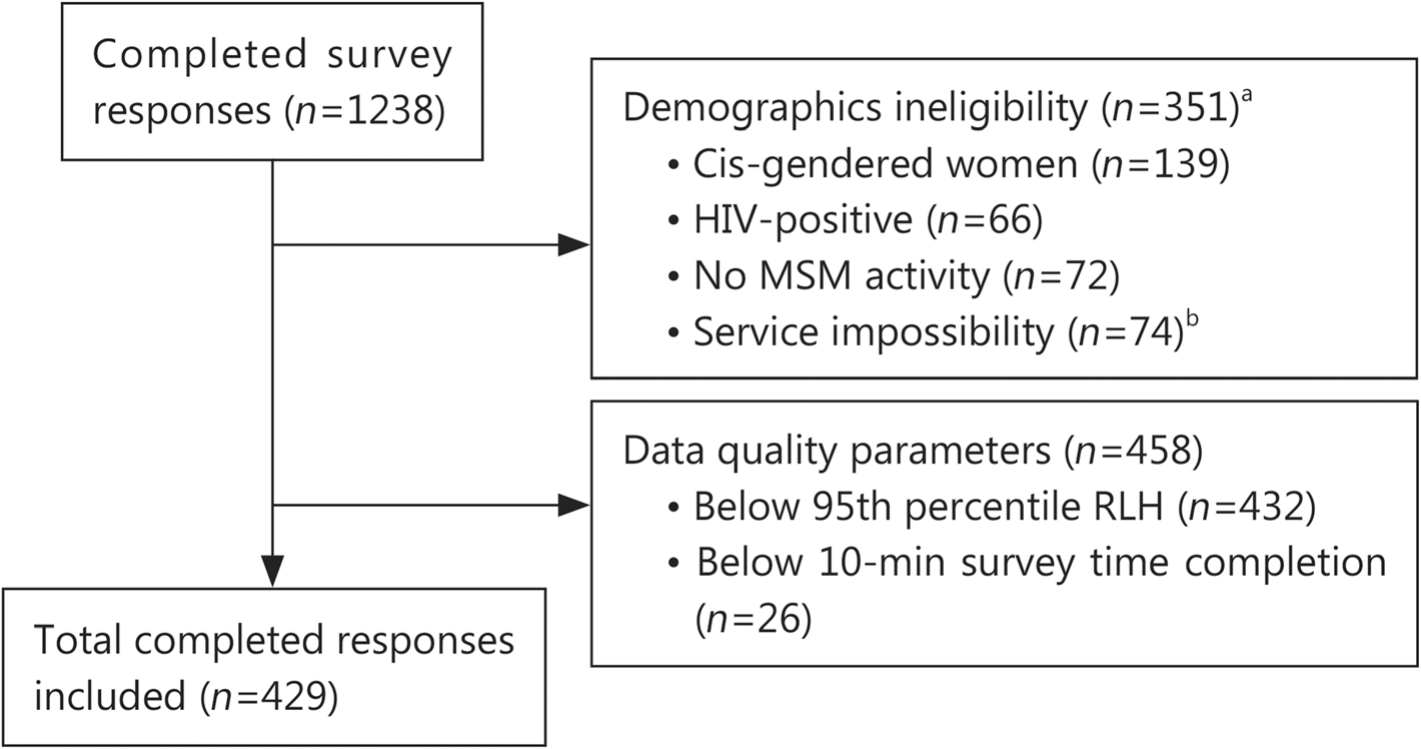
CONSORT diagram for participant enrolment and exclusion. ^a^Inclusion criteria demographics were assessed twice; at consent screen for eligibility, and again after conjoint experiment. ^b^Omitted responses indicated a service impossibility, such as self-identifying as an Air Force warrant officer (does not exist). Cis cisgender, HIV human immunodeficiency virus, MSM men who have sex with men

Respondent enrolment and exclusion are shown in CONSORT style in Fig. 1. To ensure data integrity and eliminate random or duplicate responders, security features within the Sawtooth software and servers recognize returning study participants through the use of internet browser cookies and IP addresses. It also prevents repeated or duplicate attempts to retake the survey [44]. Since extensive pilot testing required at least 10 to 15 min, responses completed in less than 10 min (or if a respondent selected the same answer for all items) were excluded. Furthermore, the root likelihood (RLH) fit statistic for each respondent was analyzed to evaluate within-respondent choice consistency. RLH, which has a probability value from 0 to 1.0, was used to discriminate between respondents who answered choice-questions consistently or randomly [45]. The survey design was tested by 1000 computer-generated mock respondents to determine the median RLH for "random responders" at the 95% percentile (0.5178 RLH). Survey respondents with an RLH below this score were excluded, as the inclusion of "random responders" can affect the calculation of preference scores and participation rates [45].

For conjoint analyses, a hierarchical bayes (HB) procedure was used to estimate part-worth utility scores (PWUS) on an individual level for accuracy and efficiency [46, 47], and was used to analyze the PWUS of the aggregated sample across all 16 attribute levels. The resulting PWUS of the levels under each attribute category are zero-centered; meaning that the sum of the level scores under each attribute category equal to zero. Scores that are further away from zero (0) indicate a stronger positive or negative preference for the level choice in relation to the other level choices under the same attribute [39, 43, 47]. After identifying each attribute level PWUS, the attribute relative importance scores (RIS) was calculated to characterize the magnitude of influence that each attribute category has on the respondent preference decision-making. The RIS for this study was calculated by dividing the range of PWUS for levels under each attribute by the sum of the ranges, and then multiplying by 100 [48, 49]. For example, an attribute RIS at 45% denotes that 45% of an individual’s decision making for program engagement is influenced by preferences within that attribute category. The PWUS were then used to predict the share of preference (participation interest) among eight hypothetical PrEP program scenarios. PrEP program scenarios were configured after a variety of currently available or hypothetical PrEP program models. For this study, participation rates for these PrEP scenarios were generated using the randomized first choice model, in which PWUS are summed across the levels corresponding to each option, and then exponentiated and rescaled, so they sum to 100 [48, 49]. This approach is based on the assumption that respondents or consumers will prefer a product with the highest composite utility (or value) adjusting for both attribute and program variability [48]. The randomized choice model accounts for variation in each participant’s total utility for each option and error in point estimates of the utility, and has been shown to have better predictive ability than other shares of preference models [49]. All data analyses were performed using XLSTAT and Sawtooth Lighthouse Studio 9.0.

## Results

### Participants

A total of 429 respondents were included in the analyses. The participants were 30 years of age on average, mostly white (72.0%) and cis-gendered male (96.7%, Table 2). 46.4% were officers, and 48.7% were in the Army (48.7%). Sixty-two point seven percent of the participants had depressive symptoms, 89.3% had high objective risk for acquiring HIV [42]. Eighty-three point nine percent of the participants reported at least one episode of condomless receptive anal sex (CRAS) within the prior six months. Thirty-six point eight percent of the participants were “somewhat” or “extremely” uncomfortable with talking about sex with their primary care providers (PCP).

**Table 2:** Characteristics of the participant demographics, stratified by PrEP experience

Index	Total sample (*n* = 429)	PrEP-experienced (*n* = 357)	No PrEP experience (*n* = 72)	*P*
Age ($\overline{x}$ ± *s*)	29.9 *±* 4.7	30.0 *±* 4.4	29.5 *±* 5.7	0.42
Gender [*n*(%)]				0.48
Male	415 (96.7)	346 (96.9)	69 (95.8)	
Trans female	11 (2.6)	8 (2.2)	3 (4.2)	
Trans male	3 (0.7)	3 (0.8)	0 (0.0)	
Race [*n*(%)]				0.46
White	309 (72.0)	253 (70.9)	56 (77.8)	
Black	78 (18.2)	70 (19.6)	8 (11.1)	
All other race	42 (9.8)	34 (9.5)	8 (11.1)	
Ethnicity [*n*(%)]				< 0.01**
Hispanic	118 (27.5)	109 (30.5)	9 (12.5)	
Non-hispanic	311 (72.5)	248 (69.5)	63 (87.5)	
Rank [*n*(%)]				0.27
Enlisted	161 (37.5)	133 (37.3)	28 (38.9)	
Officer	199 (46.4)	161 (45.1)	38 (52.8)	
Warrant	69 (16.1)	63 (17.6)	6 (8.3)	
Education [*n*(%)]				< 0.05*
High school	28 (6.5)	18 (5)	10 (13.9)	
AD or some college	169 (39.4)	147 (41.2)	22 (30.6)	
Bachelor’s degree	188 (43.8)	159 (44.5)	29 (40.3)	
Grad/Prof degree	44 (10.3)	33 (9.2)	11 (15.3)	
Military branch [*n*(%)]				0.07
Air force	65 (15.2)	47 (13.2)	18 (25.0)	
Army	209 (48.7)	181 (50.7)	28 (38.9)	
Coast guard	49 (11.4)	40 (11.2)	9 (12.5)	
Marine corps	48 (11.2)	38 (10.6)	10 (13.9)	
Navy	58 (13.5)	51 (14.3)	7 (9.7)	
Region of station [*n*(%)] ^a^				< 0.001***
Midwest	55 (12.8)	45 (12.6)	10 (13.9)	
Northeast	79 (18.4)	74 (20.7)	5 (6.9)	
South	161 (37.5)	139 (38.9)	22 (30.6)	
West	129 (30.1)	97 (27.2)	32 (44.4)	
Other/OCONUS	5 (1.2)	2 (0.6)	3 (4.2)	
Depression PHQ2 screening [*n*(%)] ^b^				0.40
≥ 1	269 (62.7)	227 (63.6)	42 (58.3)	
= 0	160 (37.3)	130 (36.4)	30 (41.7)	
HIRI-MSM risk score [*n*(%)] ^c^				0.17
≥ 10	383 (89.3)	322 (90.2)	61 (84.7)	
< 10	46 (10.7)	35 (9.8)	11 (15.3)	
# of male sex partners last 6 months [*n*(%)]				< 0.01**
0–5 partners	303 (70.6)	242 (67.8)	61 (84.7)	
6+ partners	126 (29.4)	115 (32.2)	11 (15.3)	
# CRAS within last 6 months [*n*(%)] ^d^				0.61
None	69 (16.1)	56 (15.7)	13 (18.1)	
About once/month or less	249 (58.0)	211 (59.1)	38 (52.8)	
About once/week or more	111 (25.9)	90 (25.2)	21 (29.2)	
# of HIV+ partners last 6 months [*n*(%)] ^e^				< 0.001***
0 partners	268 (62.5)	205 (57.4)	63 (87.5)	
1 or more partners	161 (37.5)	152 (42.6)	9 (12.5)	
Satisfied w/current level of HIV protection [*n*(%)]				< 0.01**
Satisfied	356 (83.0)	304 (85.2)	52 (72.2)	
Unsatisfied	73 (17.0)	53 (14.8)	20 (27.8)	
Level of comfort discussing sex life w/PCP [*n*(%)]				0.42
Extremely uncomfortable	37 (8.6)	31 (8.7)	6 (8.3)	
Mostly uncomfortable	121 (28.2)	95 (26.6)	26 (36.1)	
Mostly comfortable	209 (38.7)	179 (50.1)	30 (41.7)	
Extremely comfortable	62 (14.5)	52 (14.6)	10 (13.9)	

^a^States within the U.S. Midwest (IA, IL, IN, KS, MI, MN, MO, ND, NE, OH, SD, WI), Northeast (CT, DC, DE, MA, MD, ME, NH, NJ, NY, PA, RI, VT), South (AL, AR, FL, GA, KY, LA, MS, NC, SC, TN, VA, WV, AZ, NM, OK, TX), West (AK, CA, CO, HI, ID, MT, NV, OR, UT, WA, WY), other/OCONUS (overseas, out of country)

^b^Yes/no PHQ2 version. Scores ≥1 positive screen [41]

^c^1–47 range. Scores ≥10 defined as high risk for HIV [42]

^d^The number of condomless receptive anal sex (CRAS) within the past 6 months

^e^The number of sex partners that were HIV-positive in the past 6 months

*PrEP* pre-exposure prophylaxis, *AD* active-duty, *OCONUS* Outside Continental United States, *PHQ2* patient health questionnaire-2, *HIRI-MSM* HIV incidence risk index for men who have sex with men, *CRAS* condomless receptive anal sex, *HIV* human immunodeficiency virus, *PCP P*rimary care provider

*P < 0.05; **P < 0.01; ***P < 0.001

When stratified by PrEP experience, those with no previous PrEP experience were mostly of non-Hispanic ethnicity, stationed in the Western region of the U.S., and more likely to report having only a high school education. These participants also reported a lower number of male and HIV-positive partners within the last six months, and tended to report being less satisfied with their current level of HIV protection.

### Relative importance and part-worth utility scores

Table 3 shows the relative importance scores (RIS) of the five attributes and Table 4 shows the part-worth utility scores (zero-centered) for each attribute level. Overall, the dosing method was the most important attribute, regardless of PrEP experience. For participants reporting PrEP experience, the daily tablet was the most preferred dosing method option, followed by the on-demand tablet regimen. For those with no previous experience with PrEP, the bi-monthly PrEP injection was the most preferred dosing method option, with the yearly implant and daily tablet preferred but to a lesser degree. Among the remaining attributes, both groups generally preferred the option to see a military healthcare provider, to use a smartphone to conduct the PrEP visit, and to utilize an on-base location for laboratory evaluation and receipt of medication. Thus, respondents with no PrEP experience are less likely to select “None” compared to PrEP-experienced individuals, and are more likely to initiate PrEP regardless of program configuration.

**Table 3:** Relative importance scores (RIS)^a^ of PrEP attributes, stratified by PrEP experience (%)

PrEP program attribute	Total sample (*n* = 429)	PrEP-experienced (*n* = 357)	No PrEP experience (*n* = 72)
Dosing method	45.20	43.53	53.57
Provider type	15.80	16.39	13.13
PrEP visit location	14.50	15.15	11.44
Lab evaluation location	13.40	13.52	12.65
PrEP dispensing venue	11.00	11.41	9.21

^a^Relative importance scores reflect the influence that each attribute has on a participant’s decision-making (standardized to sum 100%)

**Table 4:** Part-worth utilities (zero-centered values)^a^ of PrEP program attributes and level choices, stratified by PrEP experience

Attributes and levels	Total sample (*n* = 429)	PrEP-experienced (*n* = 357)	No PrEP experience (*n* = 72)
Dosing method			
Daily tablet	21.75	18.85	36.13
PrEP injection	15.58	7.81	54.14
PrEP implant	14.05	8.44	41.82
On-demand regimen	8.99	13.93	−15.51
PrEP rectal douche	−60.37	−49.03	− 116.59
Provider type			
Military	5.55	6.20	2.33
Civilian	−5.55	−6.20	−2.33
PrEP visit location			
Smartphone	7.69	7.72	7.53
On-base	2.45	3.10	−0.81
Off-base	−10.13	−10.82	−6.73
Lab evaluation location			
Provide labs on-base	12.65	12.16	15.09
Provide labs off-base	−9.68	−9.09	−12.60
Home-based mail-in kit	−2.97	−3.07	− 2.49
PrEP dispensing venue			
Receive PrEP on-base	12.66	13.15	10.23
Receive PrEP off-base	−8.42	−8.89	−6.11
Receive PrEP by mail	−4.23	−4.26	−4.11
None ^b^	−54.7	−53.70	−59.63

^a^Zero-centered part-worth utility scores imply the positive or negative magnitude of the participant’s preference for the level choice in relation to the other level options within the same attribute

^b^The “None” parameter represents the positive or negative magnitude in which a respondent is likely to select “None” (not willing to take PrEP in any scenario despite program configuration)

*PrEP* pre-exposure prophylaxis

### Preferences for PrEP program scenarios

Table 5 describes the configuration of the eight PrEP programs and Table 6 displays the participation interest scores across each individual PrEP program scenario if it were available to them as an option.

**Table 5:** Description of hypothetical PrEP scenarios with different attributes and levels

PrEP scenario ^a^	Dosing method	Provider type	Visit location	Lab evaluation	Dispensing venue
On-base military daily tablet	Daily tablet	Military	On-base	On-base	On-base
Smartphone military daily tablet	Daily tablet	Military	Smartphone	On-base	On-base
Smartphone military on-demand	On-demand	Military	Smartphone	On-base	On-base
Smartphone military injection	PrEP injection	Military	Smartphone	On-base	On-base
Remote military injection	PrEP injection	Military	Smartphone	Home kit	On-base
Smartphone military implant	PrEP implant	Military	Smartphone	On-base	On-base
Off-base civilian daily tablet	Daily tablet	Civilian	Off-base	Off-base	Off-base
Off-base civilian rectal PrEP	Rectal douche	Civilian	Off-base	Off-base	Off-base

^a^Scenarios descriptions reference Scenarios 1 through 8 in Table 6

*PrEP* pre-exposure prophylaxis

**Table 6:** Participation interest (share of preference) of individual PrEP program scenarios, stratified by PrEP experience^a^ (%)

PrEP Scenario ^b^	Total sample (*n* = 429)	PrEP-experienced (*n* = 357)	No PrEP experience (*n* = 72)
On-Base military daily tablet	66.4	65.0	73.1
Smartphone military daily tablet	69.6	68.0	78.0
Smartphone military on-demand	67.6	67.6	67.4
Smartphone military injection	69.6	67.4	80.5
Remote military injection	67.9	65.8	78.3
Smartphone military implant	68.5	66.4	78.7
Off-base civilian daily tablet	57.7	56.5	63.7
Off-base civilian rectal PrEP	40.5	42.4	30.9

^a^Share of preference denotes the percent of respondents that would prefer or have interest to participate in the respective PrEP program scenario with a particular combination of program features based on utilities obtained during the conjoint survey

^b^Descriptions of PrEP scenarios 1 through 8 are explained in Table 5

*PrEP* pre-exposure prophylaxis

The total participation interest rate for the aggregate sample was 66.4% for Scenario 1 (On-Base Military Daily Tablet; the currently-available PrEP program within the military healthcare system today). Incorporating a smartphone PrEP visit feature into a military daily tablet PrEP program (Scenario 2, Smartphone Military Daily Tablet), and this program scenario resulted in a 3% increase in total participation interest rate to 69.6%. Scenario 2 also resulted in significant gain in participation interest among those with no PrEP experience (to 78.0%). Offering an on-demand tablet regimen within a smartphone-based military PrEP program (Scenario 3, Smartphone Military On-Demand) resulted in a marginal increase in the overall participation interest rate (67.6%) and a reduction in respondents with no PrEP experience (67.4%).

Programs with longer-acting PrEP options in the form of injectables and implants (Scenarios 4 through 6) were configured for military members whose personal or work-related circumstances compel the individual to seek PrEP options with fewer dosing administrations or from a remote location with limited resources. When compared to the Scenario 1, an injectable PrEP option did not alter participation interest rate in PrEP-experienced individuals, but garnered a higher participation interest rate in those with no PrEP experience (80.5%) when injectable PrEP is offered within a smartphone-based military PrEP program (Scenario 4, Smartphone Military Injection), and a participation interest rate of 78.3% when offered through a distance-based military PrEP program (Scenario 5, Remote Military Injection). Similarly, a PrEP implant offered within a smartphone-based military PrEP program (Scenario 6, Smartphone Military Implant) did not change the participation interest rate in PrEP-experienced respondents (66.4%) but resulted in a higher participation rate in subjects without experience with PrEP (78.7%).

Off-base program configurations (Scenario 7 & 8) were configured to represent civilian-equivalent, off-base, PrEP programs that circumvent the military and on-base aspects of a PrEP program. These programs had the lowest overall participation interest rate (57.7% with daily tablet PrEP program, and 40.5% with rectal douche option).

## Discussion

The current study demonstrated an overall preference for daily tablet PrEP services at an on-base location vs. civilian and off-base settings, yet those with no previous PrEP experience have a stronger preference for longer-acting injectables and implants. Additionally, over half of all respondents screened positive for depressive symptoms, the majority of respondents engage in risk behaviors that categorize them as having a high risk for acquiring HIV. Over one-third of respondents reported discomfort in discussing their sex life with PCP. With a growing body of literature suggesting a link between depression and sexual risk behaviors among MSM [50–52], it may be beneficial for PrEP-prescribing providers to provide PrEP clinics that are sensitive and inclusive to sexual identity and to remain vigilant to address factors related to mental and sexual health specific to MSM.

In the conjoint experiment, dosing method attribute was the most critical and influential preference factor within a PrEP delivery program, with a strong overall preference for a daily tablet among the total sample. Among those with no previous PrEP experience, a dominant preference for PrEP injectables and implants suggests that a demand for these longer-acting PrEP methods exists within this population if these alternatives become available. The long-acting injectable cabotegravir for PrEP has demonstrated superiority over oral tablet PrEP [53, 54], and could become further prioritized by future users as efficacy data becomes more widely known. The MK-8591-eluting PrEP implants have also achieved promising preliminary results [55], with clear benefit over daily tablet for individuals with adherence concerns or an unpredictable work schedule [56]. The expeditionary nature often entails military members to relocate, deploy, or miss regular follow-up appointments [57, 58]. Therefore, availability of longer-acting PrEP modalities should be an important component of the future PrEP programs for military members.

The current study confirmed a preference to see a military provider for PrEP services, highlighting the important role of military healthcare providers. However, a survey of military health care providers regarding PrEP knowledge and prescription habits revealed that 49% rated their knowledge as poor and only 29% had ever prescribed it [2]. Additionally, most military members receive their PrEP prescription only after seeing an infectious disease specialist [2], suggesting that military PCP may feel uncomfortable prescribing PrEP. This lower level of PrEP knowledge and prescription practice may contribute to the heterogenous nature of PrEP availability that currently seems dependent on a military member’s geographic location [2], and could explain the statistically significant difference in respondents’ regions of station within this study when stratified by PrEP experience. An increase in PrEP knowledge has been associated with an increase in prescribing habits [59], again suggesting military PCP with necessary training and resources to comfortably prescribe PrEP may help military members engage in a wider availability of PrEP services without the extra step of a referral to an infectious disease specialist. Further research is needed to explore the preference for a military provider within this context, and how this preference can best be leveraged to improve PrEP implementation.

In the current study, respondents with no previous PrEP experience reported fewer male sexual partners and fewer HIV-positive partners within the last six months, and more likely to report being unsatisfied with their current level of HIV protection. Given that sexual contact with men and condomless sex have been found to be the most common indications for initiating PrEP among military MSM [2], further studies will need to explore what type of PrEP programs could best accommodate the desire for more intimacy with male partners without using condoms.

This study has limitations. First, this study utilized self-report from a convenience sample recruited from an online social media group comprised of U.S. military members who identify themselves as LGBT. As a result, we could not verify actual eligibility (e.g., inclusion and exclusion criteria). Having said that, existing literature that compared MSM recruitment via online methods versus in-person had found similar samples of HIV/STI prevalence and HIV-testing patterns among MSM [60, 61]. Also, our findings may not be generalizable to at-risk military members who have sex with men but do not identify themselves as being MSM or LGBT. We also excluded a large number of respondents based on RLH cut-offs. There is a rising trend of “random responders” and “survey-bots” that attempt duplicate submission of surveys that provide financial compensation and can impact preference data if included within final the analyses [45]. These RLH standards follow evidence-based consistency cut-offs to eliminate these “random responders”, but could nonetheless introduce sample selection bias. Finally, reporting preferences is distinct from actual behavior. The final evidence will come only from future studies when the preference data are implemented into practice.

## Conclusion

In military members with a high risk of acquiring HIV, PrEP programs with the following features are preferred: daily tablet, injection or implant, medical visit provided by a military healthcare provider through a telehealth smartphone app, and on-base locations to provide laboratory samples and to receive PrEP medication.

## Supplementary Information

**Additional file 1.**

## Data Availability

The datasets used and/or analyzed during the current study are available from the corresponding author on reasonable request.

## Abbreviations

ACBC: adaptive choice-based conjoint
AD: active-duty
APO: Army Post Office
Cis: cisgender
CONSORT: consolidated standards of reporting trials
CRAS: condomless receptive anal sex
HB: hierarchical bayes
HIRI-MSM: HIV incidence risk index for men who have sex with men
HIV: human immunodeficiency virus
IP: internet protocol
LGBT: lesbian gay bi & transgender
MSM: men who have sex with men
OCONUS: Outside Continental United States
PHQ2: patient health questionnaire-2
PrEP: pre-exposure prophylaxis
PCP: primary care provider
PWUS: part-worth utility scores
RIS: relative importance score
RLH: root likelihood
