# Supplementary material for: Preferences for pre-exposure prophylaxis among U.S. military men who have sex with men: results of an adaptive choice based conjoint analysis study
Source: Mil Med Res. 2021 May 19;8:32. doi: 10.1186/s40779-021-00323-6 (PMC8132436; doi:10.1186/s40779-021-00323-6)
Supplement: Supplementary file 1 — Additional file 1. [file 40779_2021_323_MOESM1_ESM.docx]

| **Additional figure** Sample conjoint choice task screener item. Attribute level options presented within programs change as choice tasks are made, which results in captured preference data on the most important attributes driving a respondent’s selection of a particular program configuration. |
| --- |
| 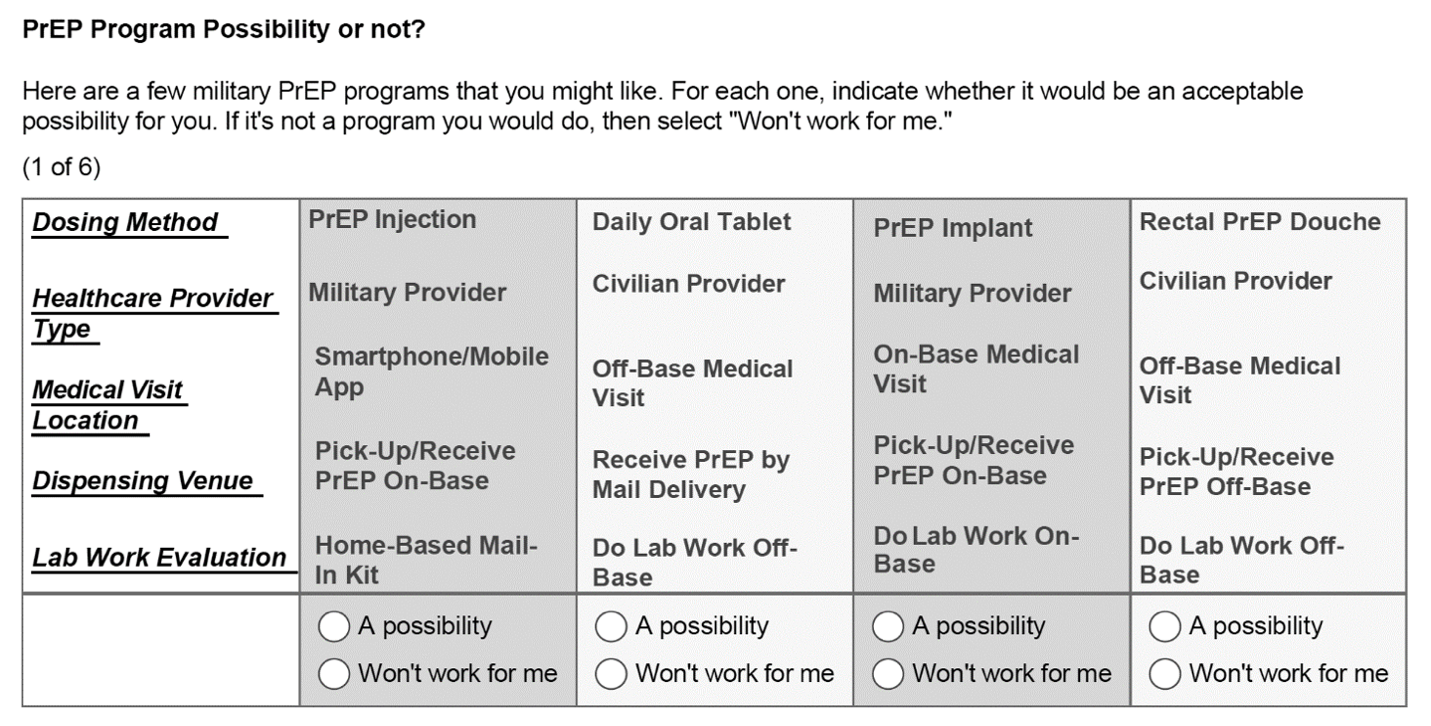 |
